# Chloramphenicol Residue Analysis in Food and Environmental Matrices: Regulatory Framework and Advances in Trace-Level Determination

**DOI:** 10.3390/molecules31091440

**Published:** 2026-04-27

**Authors:** Antonella Maria Aresta, Nicoletta De Vietro, Giovanna Mancini, Carlo Zambonin

**Affiliations:** Department of Biosciences, Biotechnologies and Environment, University of Bari Aldo Moro, via E. Orabona 4, 70125 Bari, Italy; nicoletta.devietro@uniba.it (N.D.V.); g.mancini1@phd.uniba.it (G.M.); carlo.zambonin@uniba.it (C.Z.)

**Keywords:** chloramphenicol, residue analysis, LC–MS/MS, food and environmental matrices, regulatory monitoring

## Abstract

Chloramphenicol is a broad-spectrum antimicrobial agent whose use in food-producing animals is prohibited in many countries due to its association with severe adverse effects, including idiosyncratic aplastic anemia and genotoxicity. Despite these restrictions, chloramphenicol residues continue to be detected in food products, environmental compartments, and biological matrices, highlighting the need for reliable and sensitive analytical monitoring. This review provides a comprehensive overview of current analytical strategies for the detection of drugs in food and environmental samples, covering screening and confirmatory techniques, sample preparation approaches, and regulatory aspects. Rapid screening methods, such as enzyme-linked immunosorbent assays (ELISAs), lateral flow immunoassays (LFIAs), and biosensors based on antibodies, aptamers, and molecularly imprinted polymers, enable fast and cost-effective preliminary detection. Recent advances in nanomaterials and signal amplification strategies, including fluorescent reporters and surface-enhanced Raman scattering (SERS), have significantly improved sensitivity and assay performance. However, confirmatory methods based on liquid chromatography coupled with tandem mass spectrometry (LC–MS/MS) remain the reference standard due to their superior selectivity, sensitivity, and quantitative reliability. Attention is given to sample preparation workflows, including QuEChERS-based protocols and microextraction techniques, which enable efficient analysis of complex matrices. Finally, current regulatory frameworks and analytical challenges related to zero-tolerance policies are discussed, emphasizing the importance of robust and validated analytical methods for effective monitoring and food safety assurance.

## 1. Introduction

The widespread use of antimicrobial drugs (AMDs) in livestock production began in the 1940s and progressively expanded to include both therapeutic and non-therapeutic applications. Consequently, residues of these compounds have been detected in foods of animal origin [1]. Early reports from Europe and the United States already documented antibiotic contamination in milk and meat during the 1960s [2,3].

Initial concerns were raised by the food industry, as antimicrobial residues interfered with starter cultures used in dairy processing [4]. Only later did attention shift toward public health implications, including allergic reactions and toxicological risks associated with specific molecules [5]. Today, antimicrobial use is framed within the One Health paradigm, which recognizes the interconnection between human, animal, and environmental health [6]. Within this context, chloramphenicol (CAP) represents a paradigmatic case, combining high antibacterial efficacy with severe toxicological concerns.

**Figure 1:**
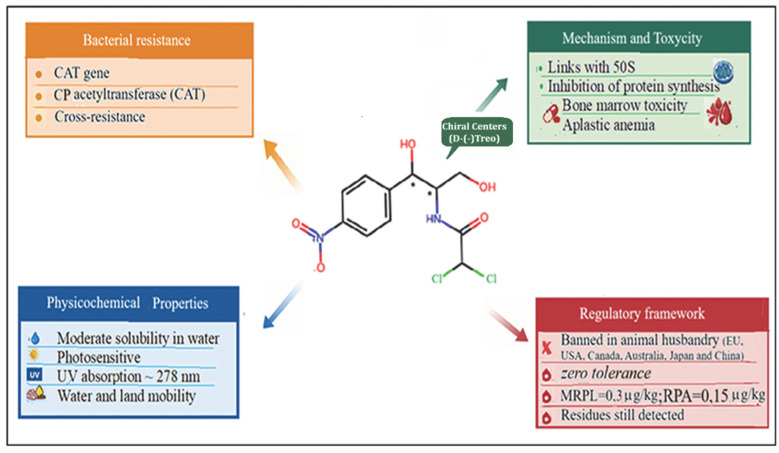
Scheme of the main chemical, biological, toxicological, and regulatory properties of CAP; CAT = chloramphenicol acetyltransferase; * = chiral center.

First isolated from *Streptomyces venezuelae* in 1947 and now produced synthetically, CAP, namely 2, 2–Dichloro-N–[β-hydroxy-α-(hydroxymethyl)-p-(nitrophenylethyl)] acetamide, is characterized by a nitro-substituted aromatic ring, an aliphatic side chain containing two chiral centers, and several polar functional groups as shown in Figure 1, which determine its physicochemical behavior, water solubility and affinity for polar organic solvents, and underlie both its biological activity and analytical detectability.

Due to its lipophilic character, CAP readily diffuses across bacterial membranes. Among its four stereoisomers, only the D-(–)-threo form is biologically active [7]. The compound inhibits bacterial protein synthesis by binding to the 50S ribosomal subunit and interfering with peptidyl transferase activity. However, CAP can also affect mitochondrial ribosomes in humans, a mechanism associated with rare but severe adverse effects such as aplastic anemia [8].

Because of its toxicological profile and the absence of a safe exposure threshold, CAP use in food-producing animals is strictly prohibited in the European Union and many other countries (USA, Canada, Australia, Japan, and China). In the EU, Commission Decision 2002/657/EC [9] established, following the absence of a maximum residue limit (MRL), a minimum required performance limit (MRPL) of 0.3 μg/kg for analytical detection. This was later replaced by the Reference Point for Action (RPA) of 0.15 μg/kg under Commission Regulation (EU) 2019/1871 [10], which has been in force since November 2022. The RPA does not represent a toxicological threshold but defines the minimum concentration that laboratories must reliably detect and confirm for regulatory enforcement.

Despite the prohibition, CAP residues continue to be reported in foods of animal origin and in environmental matrices [11,12,13,14]. The compound has been detected in surface waters, sediments, soils, and aquatic organisms, where it is considered an indicator of anthropogenic pharmaceutical contamination [15,16,17]. Environmental occurrence is attributed to illegal veterinary use, incomplete removal during wastewater treatment, and global trade of contaminated products [18,19].

In animal organisms, CAP may be excreted unchanged or metabolized to CAP-glucuronide, which can serve as an analytical marker of exposure [20,21]. Its continued detection in aquaculture products from several less developed countries further highlights the need for sensitive and reliable monitoring strategies [16,17,22,23].

The combination of severe toxicological effects, zero-tolerance regulatory policies, and persistent environmental occurrences has imposed stringent analytical requirements. Reliable detection at ultra-trace levels is therefore essential to support regulatory compliance, risk assessment, and environmental monitoring.

The aim of this review is to provide a critical and up-to-date overview (2016–2026) of analytical methods for chloramphenicol determination in food and environmental matrices. Emphasis is placed on chromatographic techniques and recent advances aimed at improving sensitivity, selectivity, and operational simplicity under current regulatory constraints.

## 2. Methodology

A structured literature search of the last decade was conducted using Scopus, PubMed, ScienceDirect, and Web of Science. Search terms included combinations of “chloramphenicol” and “analytical monitoring”, and/or “LC”, “GC”, “SPE”, “MIP”, and “SPME”. Regulatory documents regarding food, water, and sediment from FAO, EFSA, WHO, and relevant government sources were included. Studies were screened for relevance, with thematic clustering into: (i) chloramphenicol and human exposure; (ii) chloramphenicol regulatory aspects; (iii) chloramphenicol analytical determination; (iv) chloramphenicol selective clean-up. The following sections provide an up-to-date mapping of the main rapid screening and confirmation techniques, along with a discussion of their main benefits and critical aspects.

## 3. State of the Art of CAP Analytical Determination

**Figure 2:**
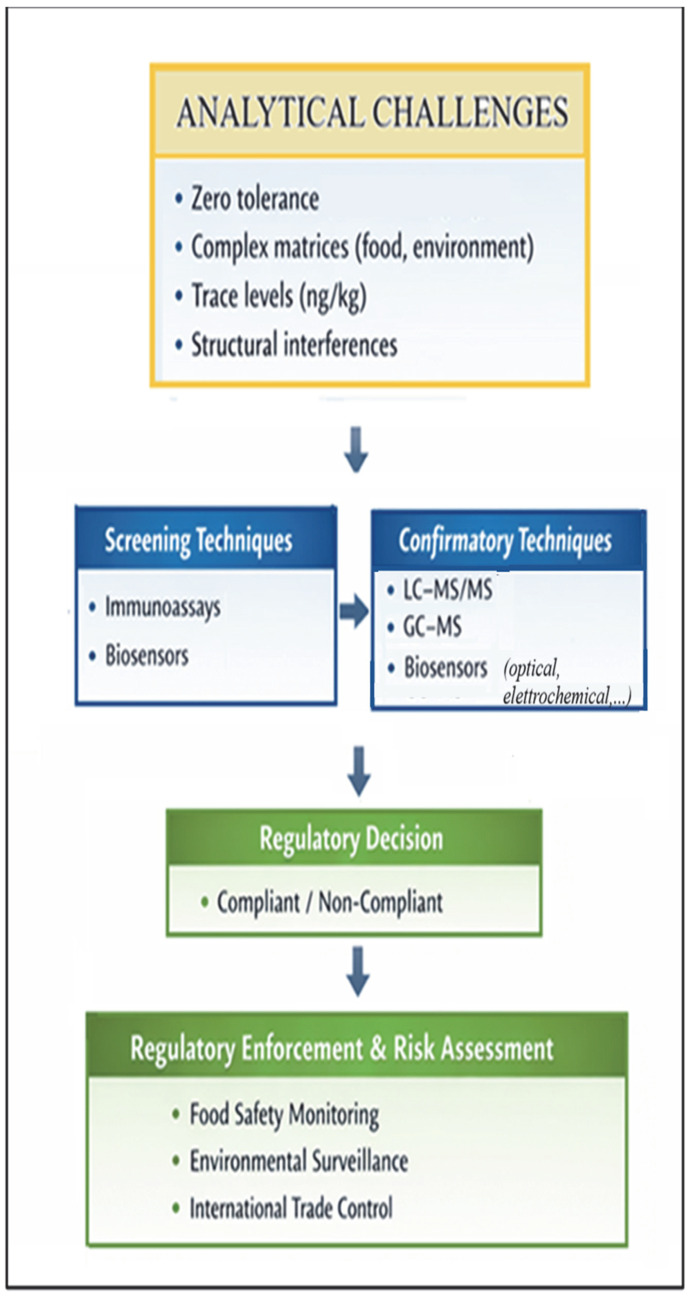
Analytical implications for CAP determination.

Chloramphenicol determination represents a particularly demanding analytical task due to the zero-tolerance regulatory framework and the requirement to reliably detect the compound at ultra-trace levels in complex food and environmental matrices. Figure 2 summarizes the main analytical implications associated with CAP monitoring.

Analytical control of veterinary drug residues follows a two-tiered approach, consisting of screening and confirmatory methods. Screening methods constitute the first level of evaluation and are designed for rapid identification of potentially non-compliant samples. They may be qualitative, semi-quantitative, or quantitative, but must be validated to ensure a false-compliant rate (β error) ≤ 5%, as established by Commission Regulation (EU) 2021/808 [24]. Suspected non-compliant results obtained during screening must be verified using a confirmatory method. Confirmatory methods, in contrast, must provide unequivocal information on the chemical identity of the analyte and enable accurate quantification. For prohibited or non-authorized substances such as CAP, confirmatory methods must demonstrate a false non-compliance rate (α error) ≤ 1%. Validation schemes in this field are therefore based on specific decision-limit parameters (CCα and CCβ), which distinguish veterinary drug residue analysis from other areas of food safety, such as pesticide residue monitoring [25]. These requirements have significantly influenced the evolution of analytical strategies for CAP determination. Over the past decade, a wide range of analytical approaches has been developed for CAP determination, differing in sensitivity, selectivity, throughput, and regulatory suitability. These methodologies, including immunochemical screening tools, confirmatory color techniques, and the latest optical and electrochemical biosensor techniques, are summarized and critically compared in Table 1.

In general, the LODs reported in Table 1 have, in most cases, been obtained under optimized experimental conditions, often using buffer solutions, diluted matrices, or strongly purified samples.

Although the analytical performance of several emerging screening approaches, including biosensors and SERS-based platforms, is increasingly competitive in terms of sensitivity, their robustness, inter-laboratory reproducibility, and regulatory acceptance remain lower than those of LC–MS/MS methods, which continue to represent the only fully validated and legally enforceable confirmatory technique under zero-tolerance frameworks. The main analytical methods for CAP determination are discussed in the following sections.

## 4. Rapid Screening Methods for CAP Detection

Rapid screening methods constitute the first analytical tier for CAP monitoring in food and environmental matrices. These approaches are based on selective molecular recognition mechanisms and are designed to rapidly identify potentially non-compliant samples prior to confirmatory chromatographic analysis.

**Figure 3:**
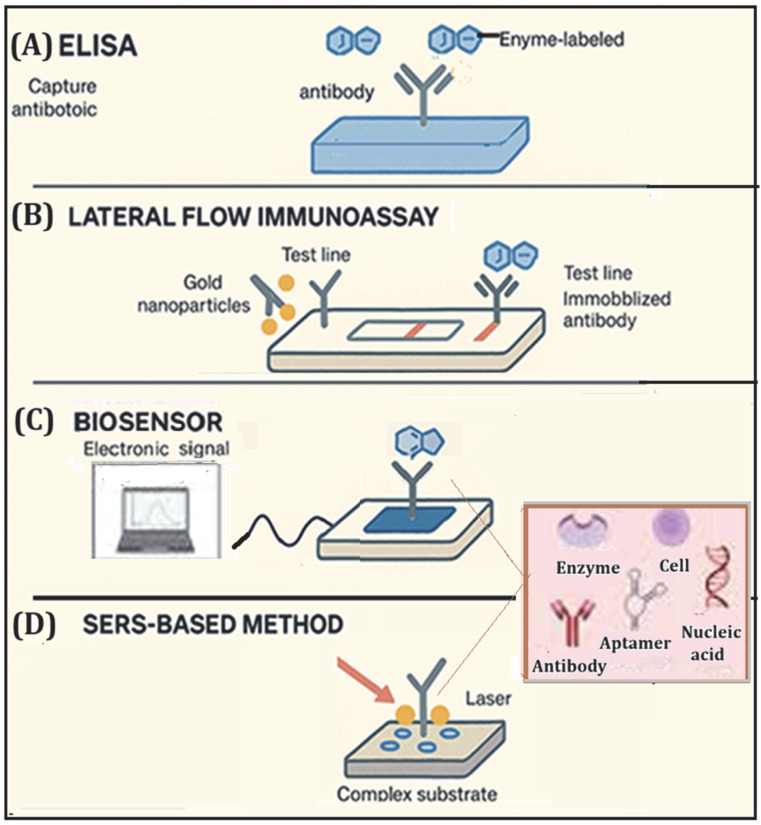
Rapid screening methods constitute the first analytical tier for CAP monitoring in food and environmental matrices. These approaches are based on selective molecular recognition mechanisms. (**A**) Enzyme-Linked Immunosorbent Assay (ELISA); (**B**) Lateral Flow Immunoassay (LFIA); (**C**) Biosensor (enzyme-based, immunosensors, aptasensors, DNA biosensors, and cell-based biosensors), employing electrical, thermal, or piezoelectric transduction mechanisms; (**D**) Surface-Enhanced Raman Scattering (SERS)-based methods.

Figure 3 schematically illustrates the principal configurations currently adopted in CAP screening devices, highlighting differences in recognition elements, signal transduction mechanisms, and analytical complexity.

Immunochemical assays, including ELISA and LFIA formats, and surface-based biosensors, which combine recognition elements with electrochemical or optical transducers, are among the most common screening methods. Advanced signal amplification strategies, such as fluorescence nanomaterials and surface-enhanced Raman scattering (SERS), can be integrated into both devices to expand their performance. The main advantages of screening methods include rapid response, operational simplicity, relatively low costs, and suitability for high-throughput monitoring. However, limitations in quantitative accuracy, potential cross-reactivity, and susceptibility to matrix effects require that suspect samples be confirmed by chromatographic techniques, typically LC–MS/MS.

### 4.1. Immunochemical Assays (ELISA and LFIA)

Competitive ELISAs remain among the most widely applied laboratory-based screening tools for CAP [80]. Due to its low molecular weight (323.13 g/mol), CAP acts as a hapten and must be conjugated to carrier proteins to elicit antibody production. In competitive formats, free CAP in the sample competes with enzyme-labeled CAP conjugates for antibody binding sites, generating an inverse relationship between signal intensity and analyte concentration.

Detection typically relies on horseradish peroxidase (HRP) with chromogenic substrates such as 3,3′,5,5′-tetramethylbenzidine (TMB), o-phenylene diamine (OPD), or 2,2′-azino-bis(3-ethylbenzothiazoline-6-sulphonic acid) (ABTS) under peroxidase conditions.

Although ELISAs provide good sensitivity and high sample flow, their analytical performance can be influenced by cross-reactivity and matrix effects. Ahmed et al. [80] highlighted that variability in antibody affinity and specificity among different manufacturers can significantly impact the reproducibility of commercial immunoassay kits, thereby affecting result comparability.

In a complementary study, Rimkus et al. [28] evaluated the ability of five commercial ELISA kits and the scintillation-based and commercial Charm^®^ II system to detect the four CAP stereoisomers in honey. These immunoassay tests are widely accepted for screening for CAP residues in food, including honey. According to this study, all investigated ELISA/Charm^®^ tests carry the risk of systematically false (false negative) results for CAP. The authors also find a discrepancy between test results and LC–MS/MS, and highlight the critical challenges associated with immunodiagnostic screening for CAP residues. Rousseau et al. [37] further assessed several commercial ELISA kits for the detection of CAP in muscle and aquaculture products. The methods were validated in accordance with European Regulation (EU) 2021/808 [24] and the European Guideline on Screening Method Validation (2023) [81]. The results of this study, on the other hand, demonstrate the suitability of immunodiagnostic tests for the tested matrices. Importantly, all kits exhibited acceptable false-positive rates and satisfactory false-negative rates (≤5%), confirming their reliability within regulatory screening frameworks.

LFIA provides a portable, on-site alternative. Conventional gold nanoparticle (AuNP) competitive LFIAs offer minute-scale results but often face sensitivity and quantitation limits in complex matrices, motivating the adoption of brighter labels and signal-amplified chemistries [80,82]. An early demonstration of fluorescence-enhanced LFIA quantification was provided by Wang et al. [26], who developed a portable fluorescence-based LFIA for the simultaneous determination of CAP with two other analogue drugs (thiamphenicol and florfenicol) in milk, achieving improved sensitivity (LOD = sub-ng/mL under optimized conditions) and reliable quantitative performance compared to colorimetric strips.

Quantum dots (QDs) and QD-nanobeads have delivered higher brightness and improved stability versus AuNPs. For CAP in milk, a QD-based LFIA was achieved on a buffer curve or diluted milk with LOD less than ng/mL within ~20 min [27,32]. In highly diluted extracts of aquatic products, a QD-nanobeads LFIA enabled quantitative detection with LOD ≈ 3.0 ng/mL and recoveries ~83–105%, outperforming two commercial AuNP kits [32].

Parallel advances in fluorescence-based strategies have explored aggregation-induced emission (AIE) nanomaterials, which provide enhanced brightness and reduced photobleaching in lateral flow formats. In particular, AIE nanobeads further increase fluorescence brightness and photostability in LFIA formats.

Elamin Elton et al. [39], in their critical review, emphasize that the limits of detection (LODs) reported in AIE-LFIA studies vary widely. These values strongly depend on the target analyte, the food matrix, and the experimental conditions. While sensitivity improvements compared to conventional AuNP-based LFIA are typically in the order of one or more magnitudes, they are not always applicable. The authors further highlight that the high signal-to-noise ratio of AIE luminogens in the aggregated state can enable lower LODs under equivalent LFIA formats, but only under carefully controlled conditions.

A recent study reported an AIE-LFIA for CAP detection in pig hair, achieving pg/kg-level LODs and a wide linear range, thereby demonstrating the suitability of AIE reporters for sensitive screening in complex matrices [34].

Beyond label strategies, colorimetric LFAs and ELISAs have been boosted using metal–organic frameworks (MOFs) as high-capacity carriers that enhance peroxidase-like activity. For CAP detection in milk, a MOF-based hybrid indirect competitive immunoassay achieved LOD = 0.006 μg·L^−1^, ~5× better than a conventional ELISA using the same immunoreagents [30]. However, it should be noted that this LOD was obtained under highly controlled experimental conditions and may not reflect routine analytical performance.

Similarly, enzyme immobilization on MOFs (e.g., horseradish peroxidase on NU-902) has enabled fast, sensitive, and economical colorimetric detection with LOD ≈ 0.018 ng·mL^−1^, validated across multiple food matrices (shrimp, eggs, milk, honey, beef, and chicken liver) [36].

LFIA architectures specifically designed for small molecules can surpass classical competitive layouts. Sotnikov et al. [31] proposed a hybrid competitive–sandwich configuration that lowered detection limits by approximately one order of magnitude in honey samples (visual LOD: 5 vs. 50 ng/mL; instrumental: 20 vs. 300 pg/mL) compared with standard competitive LFIA.

Li et al. [35] introduced a dual-mode LFIA combining colorimetric and fluorescent detection, improving robustness and reducing false-negative results. Multiplex LFIA approaches are also advancing using nanoparticles with distinct colorimetric signatures on a single test line, as well as optimized strip designs for simultaneous detection of multiple antibiotics (including CAP) in milk, highlighting their potential for practical multi-analyte screening in food matrices [33].

Finally, emerging device-integrated approaches have been explored. For example, 4D-printed redox-responsive readers for competitive ELISA (needle-bending panel meter) enable on-site, optics-free quantification of CAP with a method detection limit (MDL) ≈ 2.5 pg·mL^−1^. These systems have been validated across a range of food matrices, including salmon, shrimp, white meat, pork, honey, and milk [38].

### 4.2. Surface-Based Biosensors

Biosensor platforms for CAP detection combine a selective recognition element, antibody, aptamer, or molecularly imprinted polymer (MIP), with a physical transducer capable of converting the binding event into a measurable electrochemical, optical, or piezoelectric signal. Unlike LFIA systems, these platforms rely on immobilized recognition layers and generally provide improved quantitative control.

Electrochemical immunosensors are among the most extensively investigated configurations. A recent review by Howlader et al. [83] summarized advances in electrochemical CAP sensors, noting that nanomaterial engineering and surface functionalization strategies have enabled detection limits at or below the ng/kg level.

Nanostructured materials have been instrumental in enhancing analytical performance. El-Moghazy et al. [41] reported a label-free electrochemical immunosensor based on a nanofibrous membrane that enabled direct analysis of milk samples with minimal pretreatment and sub-ng/mL sensitivity (LOD = 0.0047 ng/mL). A broader overview of electrochemical immunosensors for antibiotic detection, including CAP applications, was provided by Pollap and Kochana [84], who critically discussed signal amplification strategies and challenges related to real sample analysis.

More recently, surface-engineered electrochemical sensors not relying on biological recognition elements have been proposed. For instance, Švalova et al. [47] developed a voltammetric sensor based on a glassy carbon electrode modified with an electrodeposited 3,6-diethynyl-9H-carbazole functional layer, achieving sensitive and selective CAP determination via surface-mediated electrochemical interactions. Aptamer-based biosensors offer high chemical stability and tunable binding properties. El-Khoshbin et al. [43] demonstrated competitive electrochemical aptasensors for CAP detection, highlighting their potential as portable and low-cost alternatives to antibody-based systems. The structural flexibility of aptamers facilitates integration into miniaturized and multiplexed sensing platforms. Beyond electrochemical formats, aptamer-based colorimetric assays have also been explored. Tao et al. [29] critically evaluated specific and nonspecific binding phenomena in an aptamer-based colorimetric platform for CAP detection, highlighting how binding artifacts can impact assay accuracy and the need for rigorous optimization in aptasensor development.

MIP-based biosensors represent synthetic recognition systems characterized by robustness and long-term stability. Zhang et al. [45] developed a nanocomposite ZnO/C PEC-MIP photoelectrochemical sensor, with an LOD of 5.08 pg/mL, capable of selective CAP detection under variable environmental conditions, emphasizing the suitability of synthetic receptors for field applications. Further confirming the analytical value of MIP-based architectures, Wu et al. [46] developed a highly sensitive molecularly imprinted electrochemical sensor using a MIP/PAN/AuNPs RGO CS modified electrode, achieving ultra-low detection limits (LOD = 63 pM, ≈20 pg/mL) and demonstrating excellent selectivity, stability, and recovery in milk and egg samples. These results highlight the scalability and real sample applicability of MIP–electrode systems for CAP monitoring.

Tomassetti et al. [42] proposed a direct methanol fuel cell (DMFC) modified with alcohol dehydrogenase to promote the electrochemical oxidation of CAP; this system offered a simple analytical procedure and a rapid response time (approximately 25 min), demonstrating potential for pharmaceutical formulations rather than complex food matrices. In the same work, a conventional competitive immunosensor was also evaluated and showed inferior performance in terms of both sensitivity and analysis time.

Martini et al. [44] compared two competitive electrochemical immunosensor architectures, demonstrating how surface configuration and signal generation strategy significantly influence detection limits and dynamic range.

In conclusion, recent optical and electrochemical biosensors that exploit signal modulation mechanisms without the use of enzymes or antibodies (Figure 3) are effective alternatives to conventional immunochemical methods, offering reduced analysis time, lower operating costs, and simplified procedures. Zou et al. [48] have recently proposed an electrochemical platform for the detection of fluorescence based on the product catalysis of the tetrahedral DNA system and on the fluorescent extinction of MIL-101 (Fe), capable of detecting CAP directly from real matrices with minimal sample preparation and analysis times, typically less than 30 min, and with detection limits comparable to immunochemical assays. From an operational point of view, the platform can also be used both as a high-sensitivity screening tool and as a quantitative method when accompanied by linear calibration curves and adequate quality controls. Collectively, biosensor-based strategies, while not yet replacing the confirmation techniques required by the standard (LC MS/MS), represent an important evolution towards portable, fast, and user-friendly systems, useful for pre-selection in the field or in laboratories with limited resources.

### 4.3. SERS-Integrated Detection Strategies

Unlike the biosensor platforms described above, where sensitivity improvements mainly arise from advances in recognition layers and electrochemical or optical transducers, surface-enhanced Raman scattering (SERS) should be considered primarily as a signal transduction and amplification strategy rather than a standalone recognition approach. SERS can be integrated into LFIAs or employed as the optical readout in surface-based biosensors, where its exceptional electromagnetic enhancement significantly boosts analytical sensitivity.

SERS exploits plasmonic nanostructures capable of generating highly localized electromagnetic fields (“hot spots”), which dramatically amplify the Raman signal of analyte or reporter molecules, enabling ultraspecific vibrational fingerprints.

A comprehensive overview of plasmonic architectures, particularly Au–Ag bimetallic nanoparticles, which offer superior enhancement factors, improved stability, and denser hot spot formation compared to monometallic substrates, was provided by Yu et al. [85], highlighting their suitability for food contaminant detection and next-generation SERS platforms. Further supporting this, Awiaz et al. [50] reviewed the design principles and performance of Au@Ag core–shell SERS biosensors, discussing how shell thickness, morphology, and aspect ratio influence electromagnetic enhancement and overall analytical performance, providing essential guidelines for engineering high-efficiency SERS substrates.

In biosensor configurations, SERS serves as a highly sensitive optical transduction mechanism for surface-immobilized recognition layers (antibodies, aptamers, or MIPs), enabling ultra-trace detection via synergistic coupling between selective molecular recognition and plasmon-enhanced Raman amplification. The recent evolution of nanostructured substrates, including Au–Ag core–shell materials, hierarchical plasmonic architectures, and flexible SERS sensors, has positioned SERS-integrated biosensing as a powerful emerging platform for next-generation monitoring [80,85].

When incorporated into LFIA formats, SERS-active nanoparticles can substantially improve analytical sensitivity without compromising portability. Zhao et al. [51] developed a comprehensive SERS–LFIA platform that combines the two technologies with a support vector machine (SVM) model for data analysis. The device was used to detect CAP residues in aquatic products. So, CAP-specific antibody-modified gold nanostars (Au NSs) were synthesized. The detection of the analyte obtained was ultra-sensitive (LOD = 10 pg/mL; theoretical LOD = 1.6 pg/mL), clearly higher than commercial strips and without requiring sample pretreatment.

Also, in biosensor configurations, SERS serves as a highly sensitive optical transduction mechanism for surface-immobilized recognition layers (antibodies, aptamers, or MIPs), enabling detection at ultra-trace levels through synergistic coupling between selective molecular recognition and plasmon-enhanced Raman amplification. The recent evolution of nanostructured substrates, including Au–Ag core–shell materials, hierarchical plasmonic architectures, and flexible SERS sensors, has positioned SERS-integrated biosensing as a powerful, emerging platform for next-generation chloramphenicol monitoring [80,85].

As with LFIA, SERS can also be implemented on flexible substrates for direct sample interrogation. Yang et al. [40] developed a competitive immunosensor by integrating magnetic separation with SERS-based signal readout, thereby improving selectivity in complex matrices. Alternative biosensing concepts were also explored. Li et al. [49] designed a flexible paper-based SERS sensor employing flower-like silver nanoparticles, combined with AI-assisted chemometric modeling, achieving pg-level discrimination of CAP in food matrices and demonstrating the potential of AI-enhanced SERS analytics for rapid field deployment.

However, the excellent sensitivity achieved by SERS-based methods should be balanced against practical limitations affecting routine applicability. Signal reproducibility is strongly influenced by substrate fabrication, surface chemistry, and matrix-induced interferences, which may compromise quantitative robustness in complex samples. These aspects highlight the need for improved standardization and validation before widespread adoption of SERS platforms for routine residue monitoring.

### 4.4. Critical Considerations on Rapid Screening Methods

In conclusion, despite the impressive sensitivity reported in many experimental studies, the practical implementation of advanced screening platforms remains challenging in terms of substrate reproducibility, large-scale fabrication, signal standardization, and instrumentation requirements. Factors such as matrix complexity, antibody specificity, batch-to-batch reproducibility, and long-term stability of nanostructured labels often limit routine application in official control laboratories. Consequently, while biosensors and advanced LFIA systems demonstrate strong potential, only a limited number of formats have reached a level of robustness compatible with regulatory screening programs. Although these results demonstrate the operational effectiveness of screening assays, confirmatory LC–MS/MS methods remain indispensable for regulatory decision-making [86].

This regulatory suitability was further demonstrated by a proficiency test organized by the Italian National Reference Laboratory for Residues, which assessed the performance of official laboratories for chloramphenicol determination in honey at the Action Reference Point (RPA) of 0.15 µg/kg [87].

**Figure 4:**
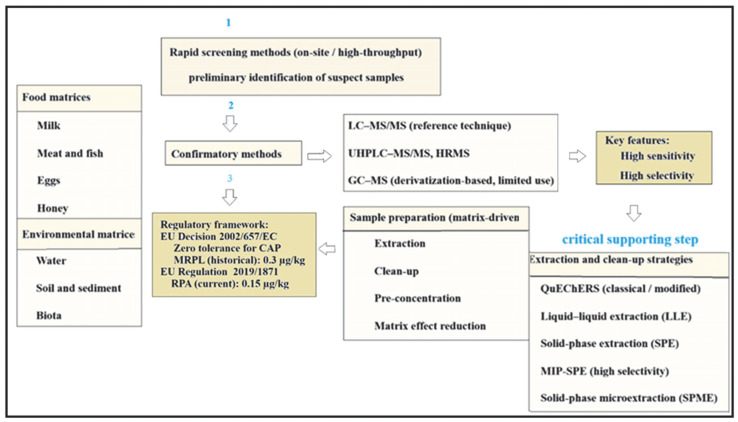
Conceptual overview of the analytical workflow for CAP determination. Rapid screening methods enable high-throughput identification of suspect samples but do not provide legally enforceable results. Confirmatory chromatographic techniques ensure definitive identification and quantification in compliance with zero-tolerance regulatory requirements. The numbers in the figure indicate the sequence of the analytical steps.

Figure 4 shows a conceptual overview of the analytical workflow for CAP determination.

## 5. Chromatographic Methods for CAP Detection

Confirmatory methods represent the highest level of analytical assurance for CAP detection and are indispensable for regulatory decision-making. Unlike screening assays, they must provide unequivocal identification and accurate quantification at trace levels, in full compliance with the stringent requirements applied to banned veterinary drugs.

Reversed-phase LC remains the most widely applied separation strategy for CAP. Conventional C18 stationary phases ensure adequate retention and symmetrical peak shape for this moderately polar compound (logP ≈ 1.1). Mobile phases typically combine water with methanol or acetonitrile [23,88,89]. While CAP generally does not suffer from severe co-elution, proper chromatographic separation is essential, especially in complex matrices such as meat, fish, honey, and sediments. Gradient elution is therefore commonly used to efficiently remove late-eluting components, maintain column cleanliness, and ensure compatibility with multi-residue workflows [88].

More specialized strategies have also been proposed. Demir and Aydoğan [21] developed a graphene-oxide-modified monolithic nano-column for ProFlow Nano LC, enabling ultra-sensitive quantification of CAP and its metabolite (CAP-glucuronide) in milk and honey. Despite excellent performance, this highly specialized system remains unsuitable for routine laboratory implementation.

### 5.1. Chromatographic Methods with Optical Detection

Historically, instrumental confirmatory analysis began with liquid chromatography coupled to ultraviolet or diode-array detection (LC–UV/DAD) and fluorescence detection (LC–FLD) [88].

In general, chromatographic techniques based on optical detectors benefit from simple tools and low operating costs. LC–FLD offers superior sensitivity compared to UV, but derivatization of compounds is required. Xu et al. [57] developed a naphthalene diimide-based precolumn derivatization procedure for the determination by HPLC-FLD of genotoxic aromatic aldehydes in pharmaceutical preparations. The procedure was successfully applied to eye drops at CAP. However, by increasing the analytical complexity of samples, more complex sample preparation procedures are required, and derivatization introduces additional variability. Therefore, even LC-FLD in complex food and environmental matrices shows insufficient extensibility compared to the current zero-tolerance regulatory requirement for CAP.

In recent years, the use of advanced microextraction techniques has significantly enhanced the effectiveness of UV/DAD-based methods, as well as supporting the eco-sustainability of sample preparations. Teglia et al. [90] show that chemometric analysis is crucial in comparing dispersive techniques for antibiotics in eggs, as it allows for choosing the most suitable technique based on the chemical–physical properties of the analytes, ensuring rapid and solvent-efficient extractions. Among these, magnetic solid-phase extraction (MSPE) [52] and magnetic ionic liquid-based liquid–liquid microextraction (MIL LLME) [53] stand out as complementary approaches, relying respectively on magnetic solid sorbents and magnetic ionic liquids as extraction phases. Additional strategies such as deep eutectic solvent magnetic nanofluids (MNF DLLME) [54], fabric phase sorptive extraction (FPSE) [55], and MOF-based sorbents [56] have also been proposed, enabling improved precision but still falling short of the requirements for regulatory confirmatory analysis.

Overall, optical detection techniques are best regarded as complementary or matrix-specific tools, whereas mass spectrometry-based LC methods constitute the cornerstone of confirmatory analysis for chloramphenicol residues, offering the selectivity and sensitivity required under the current regulatory framework.

### 5.2. Mass Spectrometry-Based Chromatographic Methods

The intrinsic limitations of optical detection systems, particularly under ultra-low regulatory limits and in complex matrices, have driven the transition toward mass spectrometry-based chromatographic techniques, which currently represent the cornerstone of confirmatory analysis for chloramphenicol residues.

#### 5.2.1. Gas Chromatography–Mass Spectrometry (GC–MS)

GC–MS has been applied to CAP determination mainly in earlier studies and in specific research contexts. Due to its low volatility and thermal instability, CAP requires chemical derivatization, most commonly silylation, to be amenable to GC analysis. Under appropriate conditions, the molecule is converted into a doubly silylated derivative. Because CAP contains two chlorine atoms, highly selective electron capture-based detection techniques, particularly GC–MS operating in negative chemical ionization (NCI) mode, provide excellent sensitivity, with reported detection limits in the sub ng/kg range for food matrices [60].

In NCI mode using methane as the reagent gas, the di TMS derivative generates a stable molecular ion at *m*/*z* 466 with a characteristic chlorine isotope pattern. Fragmentation is limited, and higher mass adducts mainly arise from interactions with methane-derived ions. This simplified spectral profile enables very high sensitivity, with absolute detection limits approaching 0.2 pg when monitoring multiple ions.

In the last decade, however, only a few studies have applied GC–MS to CAP under regulatory-compliant conditions. Rejtharová et al. [58] validated a GC–MS NCI method in accordance with Commission Decision 2002/657/EC to investigate CAP depletion in chicken muscle following controlled oral administration, achieving a decision limit (CCα) of 0.05 ng g^−1^ and demonstrating the persistence of low-level residues for up to 35 days. A subsequent feeding study [59] using the same analytical approach detected residues only at the highest feed contamination levels, supporting the hypothesis that measurable CAP residues in edible tissues are more consistent with illegal administration than with natural background exposure.

Although GC–MS provides high selectivity and sensitivity, its dependence on derivatization, longer analytical workflows, and reduced compatibility with multi-residue methods have limited its applicability in routine monitoring. For these reasons, GC–MS has been largely superseded by liquid chromatography combined with mass spectrometry detectors, which today represents the reference confirmatory technique for CAP analysis.

#### 5.2.2. Liquid Chromatography–Mass Spectrometry (LC–MS/MS, LC-Orbitrap, LC-QTOF)

Liquid chromatography coupled with mass spectrometry represents the reference analytical approach for CAP determination, enabling reliable identification and quantification at ultra-trace levels under zero-tolerance regulatory frameworks. Both triple quadrupole LC–MS/MS and high-resolution mass spectrometry (HRMS) platforms, including Orbitrap and quadrupole time-of-flight (QTOF) instruments, can achieve the decision limits and identification criteria established for regulatory control. However, their analytical roles differ substantially in terms of quantitative robustness, selectivity, and operational applicability. Triple quadrupole LC–MS/MS remains the technique of choice for routine regulatory quantification of CAP at ultra-trace levels due to its superior sensitivity and robustness in targeted MRM acquisition.

CAP is most analyzed using electrospray ionization operated in negative mode (ESI^−^), owing to the presence of phenolic and nitro functional groups that facilitate deprotonation. Under these conditions, CAP readily forms a stable deprotonated molecular ion [M–H]^−^ at *m*/*z* 321, ensuring efficient ionization and reproducible signal intensity [23].

Tandem mass spectrometric detection is typically performed in multiple reaction monitoring (MRM) mode, which represents the reference acquisition strategy employed in regulatory confirmatory analysis, allowing highly selective identification through monitoring of characteristic precursor-to-product ion transitions. The most frequently reported product ions include *m*/*z* 152 and *m*/*z* 194, corresponding to fragmentation of the aromatic ring and side chain structures. According to current European regulatory guidelines, the use of at least two MRM transitions, combined with retention time matching and ion ratio evaluation, ensures reliable confirmatory identification even at ultra-trace levels (<0.1 µg/kg). Quantitative performance is further improved with isotopically labeled internal standards, such as CAP-d_5_, which effectively compensate for matrix-induced ion suppression or enhancement and instrumental variability. This isotopic dilution approach is strongly recommended in regulatory workflows, as it effectively compensates for matrix effects and instrumental variability, significantly improving method accuracy, precision, and robustness [23,87].

The triple quadrupole LC–MS/MS remains the preferred technique for confirmatory determination in official control laboratories. Its targeted acquisition mode maximizes signal-to-noise ratio and quantitative reliability, ensuring compliance with regulatory performance criteria for confirmatory analysis.

Simultaneous determination of CAP together with chemically different compounds in multi-residue methods presents an analytical challenge, especially when the analytes differ in polarity, stability, and ionization behavior. In this context, the Orbitrap and QTOF instruments have attracted increasing interest due to their high mass accuracy, full-scan acquisition capability, and potential for retrospective data analysis.

These advances reflect the continuing evolution of LC-MS toward more versatile analytical systems capable of improving sensitivity, selectivity, and analytical flexibility in residue analysis [91]. Several studies have demonstrated the suitability of HRMS platforms for multi-residue determination of veterinary drugs and structurally related compounds, including CAP, particularly in complex matrices where conventional targeted methods may be limited [75,92]. Recent applications further highlight the growing role of HRMS in comprehensive food safety monitoring, where high selectivity and full-scan acquisition enable broader contaminant surveillance strategies [79]. HRMS has also proven valuable in biomonitoring applications, where the identification of metabolites is essential. Pastor-Belda et al. [20] developed a dispersive liquid–liquid microextraction (DLLME) method coupled with LC–QqTOF-MS for the determination of amphenicols and their glucuronide metabolites in urine, demonstrating that CAP is predominantly excreted as conjugated forms. Similarly, QuEChERS-based extraction combined with LC–QqTOF-MS has been successfully applied to environmental biomonitoring in benthic organisms, highlighting the capability of HRMS to support comprehensive residue surveillance in complex biological systems [77].

Despite these advantages, HRMS presents important limitations when applied to routine confirmatory analysis at regulatory concentration levels. Comparative studies have shown that quantitative performance parameters such as linearity, repeatability, and signal stability may be less robust than those achieved with triple quadrupole instruments, particularly at ultra-trace concentrations required for regulatory compliance [72]. Furthermore, full-scan acquisition distributes ion currents across a broader mass range, which may reduce sensitivity compared with the highly selective MRM acquisition mode used in triple quadrupole systems.

Consequently, HRMS is currently best regarded as a complementary analytical tool rather than a replacement for triple quadrupole LC–MS/MS in regulatory confirmatory analysis. While LC–MS/MS remains the gold standard technique for quantitative determination of CAP, HRMS provides significant advantages for multi-residue screening, suspect analysis, and comprehensive contaminant profiling.

Regardless of the mass spectrometric platform employed, matrix effects remain a major analytical challenge in CAP determination. Co-extracted matrix components may significantly affect ionization efficiency, leading to signal suppression or enhancement. Therefore, appropriate sample preparation, use of isotopically labeled internal standards, and matrix-matched calibration remain essential to ensure accurate quantification and compliance with regulatory requirements.

## 6. Sample Preparation and Clean-Up Strategies in LC-MS/MS

Sample preparation represents a critical step in the reliable determination of drugs at ultra-trace levels. Although LC–MS/MS provides excellent sensitivity and selectivity, analytical performance remains strongly dependent on matrix composition. Co-extracted endogenous compounds, including proteins, lipids, sugars, pigments, and humic substances, may interfere with ionization efficiency, leading to signal suppression or enhancement and compromising quantitative accuracy. Consequently, the selection of extraction and clean-up strategies is primarily dictated by matrix characteristics rather than instrumental detection capabilities.

Food and environmental matrices relevant to CAP monitoring vary widely in their physicochemical properties, as well as in viscosity and particulate load. These differences directly influence analyte recovery, the extent and direction of matrix-induced ion suppression or enhancement, and overall method robustness. As a result, matrix-specific sample preparation workflows have been developed to balance analytical reliability, sensitivity, and laboratory throughput (Table 2). In this context, more selective clean-up strategies generally lead to improved control of matrix effects by limiting the co-extraction of ion-active components, while isotopically labeled internal standards are used to compensate for residual matrix-induced signal variability at the detection stage.

### 6.1. High-Protein and High-Lipid Matrices

Matrices such as milk, meat, fish, eggs, and aquatic biota contain high levels of proteins and lipids, which represent major sources of matrix effects in LC–MS/MS analysis. These components can co-extract with CAP and interfere with ionization efficiency, leading to signal suppression or enhancement. Therefore, sample preparation typically involves protein precipitation, organic solvent extraction, and selective clean-up procedures to improve analytical reliability.

Liquid–liquid extraction (LLE) remains one of the simplest and most widely applied approaches, particularly for milk analysis. Jadhav et al. [93] developed a unified extraction strategy combining protein precipitation and salting-out partitioning, enabling simultaneous determination of 78 veterinary drugs and pesticides. Similarly, Muti et al. [66] successfully applied LLE for the multi-residue determination of CAP and other antibiotics in milk using LC–MS/MS, achieving satisfactory recoveries and robust analytical performance. Their approach demonstrated improved efficiency compared to conventional QuEChERS-based workflows, highlighting the importance of optimizing extraction chemistry for complex biological matrices.

Egg matrices present comparable analytical challenges due to their high protein and lipid content. Simple organic solvent extraction using ethyl acetate or acetonitrile has been shown to provide efficient analyte recovery and compatibility with LC–MS/MS detection. Xie et al. [94] reported recoveries exceeding 90% for CAP using ethyl acetate–acetonitrile ammonium hydroxide (49:49:2, *v*/*v*) and defatted with hexane saturated with acetonitrile. Therefore, the extraction procedure uses simple solvents that have been proven to be suitable for routine monitoring.

Alternative techniques such as accelerated solvent extraction (ASE) have also demonstrated advantages. Wang et al. [95] compared ASE with conventional LLE for CAP determination in eggs and found that ASE improved extraction speed, reduced solvent consumption, and enhanced method robustness.

To improve clean-up efficiency and reduce sample preparation time, modified QuEChERS protocols have been increasingly adopted. Ji et al. [65] proposed an innovative clean-up approach using melamine sponge as an adsorbent material, achieving rapid removal of matrix interferents while maintaining acceptable analyte recoveries. Melekhin et al. [96] developed a magnetic hyper-crosslinked polystyrene composite (HCP/Fe_3_O_4_) for selective extraction and clean-up prior to LC–MS/MS analysis. This material demonstrated excellent extraction efficiency and was successfully applied to real samples containing veterinary drug residues. Compared with conventional dispersive SPE sorbents, this approach provided comparable or improved clean-up performance while significantly simplifying workflow and reducing processing time.

For solid animal tissues such as meat and fish, classical or modified QuEChERS extraction using acetonitrile, combined with partitioning salts and dispersive clean-up using MgSO_4_, PSA, C18, or PRiME HLB sorbents, represents the most widely adopted strategy [64,68,71]. These workflows offer high sample throughput, good recoveries, and excellent compatibility with LC–MS/MS detection. However, their broad applicability inherently involves a compromise between selectivity and universality, as no single sorbent effectively removes all matrix interferents across diverse analyte classes.

To overcome these limitations, more selective solid-phase extraction (SPE) approaches have been developed specifically targeting phenicol antibiotics. Zhang et al. [97] reported an aptamer-functionalized magnetic mesoporous carbon sorbent providing high selectivity toward CAP and achieving extremely low detection limits across multiple matrices, including serum. Similarly, Wu et al. [67] developed CNW-Si-based SPE cartridges for the selective extraction of CAP and related metabolites, achieving excellent sensitivity suitable for regulatory applications. While these targeted approaches significantly reduce matrix interferences and improve analytical sensitivity, their specificity limits applicability in broad multi-residue screening workflows.

Overall, QuEChERS-based methods remain the preferred compromise between analytical performance, simplicity, and throughput in routine multi-residue analysis, whereas selective SPE approaches offer superior sensitivity and robustness for confirmatory CAP determination at ultra-trace levels.

### 6.2. Sugar-Rich Matrices

Honey represents one of the most challenging matrices for CAP analysis due to its high sugar content, viscosity, and complex chemical composition. These properties complicate analyte extraction and promote matrix effects during LC–MS/MS analysis, requiring efficient clean-up to ensure reliable quantification.

Salting-out assisted liquid–liquid extraction has emerged as a rapid and cost-effective solution for honey analysis. Rizzo et al. [98] developed a simple and economical UHPLC–MS/MS method based on salting-out extraction, achieving accurate CAP determination suitable for routine monitoring. This approach enhances analyte partitioning into the organic phase while minimizing co-extraction of highly polar sugar components.

More selective clean-up strategies have also been explored to improve analytical reliability. Molecularly imprinted polymer SPE (MIP-SPE) has proven particularly suitable for honey analysis due to its molecular recognition capability. Lowmunkhong et al. [62] reported a confirmatory LC–MS/MS method incorporating MIP-SPE, demonstrating efficient removal of sugar interferences and improved selectivity for CAP determination.

Recent advances in sorbent materials have further enhanced clean-up efficiency in multi-residue workflows. Multi-residue workflows combining optimized solvent extraction and tailored clean-up strategies have demonstrated good performance in honey and other sugar-rich matrices [14,70].

Overall, effective sample preparation in honey analysis requires a balance between efficient analyte recovery and removal of interfering sugars. While salting-out extraction provides simplicity and suitability for routine monitoring, more selective approaches such as MIP-SPE and advanced magnetic sorbents offer superior clean-up performance and improved analytical robustness, particularly for confirmatory analysis.

### 6.3. Low-Complexity Matrices

Relatively clean aqueous matrices, such as drinking water, generally require simpler sample preparation procedures. Direct injection into LC–MS/MS systems may be feasible when analyte concentrations are sufficiently high, and matrix effects are minimal. However, for ultra-trace determination, preconcentration using SPE remains the preferred approach, improving detection limits and analytical reliability [13]. Most recently, Sun et al. [99] propose salting-out assisted liquid–liquid extraction for the simple and rapid determination of veterinary antibiotic residues in aquatic products from HPLC-MS/MS.

### 6.4. Environmental and Biological Matrices

Environmental matrices such as soil, sediment, and aquatic organisms present significant analytical challenges due to their heterogeneous composition and high content of organic matter, pigments, and lipids. These components can strongly influence analyte extraction efficiency and ionization behavior.

Modified QuEChERS protocols have emerged as effective solutions for environmental monitoring applications. Acosta-Dacal et al. [100] and Shi et al. [69] demonstrated that optimized QuEChERS workflows enable efficient extraction of antibiotic residues from environmental solids while reducing solvent consumption and processing time. Similar approaches have been successfully applied to aquatic organisms. Kazakova et al. [101] reported effective recovery of antibiotics from fish tissues using ultrasound-assisted QuEChERS extraction combined with dispersive SPE clean-up. Manjarrés et al. [75] further improved extraction performance through hybrid clean-up sorbents, enhancing analytical reliability in aquaculture matrices.

Biological matrices such as urine also represent valuable tools for exposure assessment and regulatory monitoring. Chiesa et al. [72] demonstrated the applicability of LC–MS/MS for CAP determination in swine urine and muscle, highlighting the utility of urine analysis as a non-invasive monitoring approach. Similarly, Mizobe et al. [63] confirmed the suitability of UHPLC–MS/MS for trace-level detection of veterinary drugs in biological samples.

### 6.5. Critical Considerations on Sample Preparation Strategy

The choice of sample preparation strategy critically influences analytical sensitivity, selectivity, and regulatory compliance. Each technique offers specific advantages and limitations, particularly in relation to matrix complexity and confirmatory requirements (Table 3).

Liquid–liquid extraction (LLE) often involves complex workflows and lacks the robustness required for routine regulatory analysis. Enhanced performance can be achieved through ionic liquid-assisted LLE or deep eutectic solvent systems, which improve preconcentration and sensitivity with optical detectors or LC–MS/MS. Although these approaches can deliver high enrichment factors and low detection limits, their operational complexity still limits their suitability for routine regulatory applications [53,55].

QuEChERS-based approaches are broadly adopted in multi-residue workflows for meat, fish, and poultry because of their simplicity, low solvent consumption, and high throughput. Modified QuEChERS protocols remain popular for their efficiency and compatibility with multi-residue analysis [102].

Conventional SPE remains widely used for CAP clean-up prior to LC–MS/MS, offering a practical balance of simplicity, robustness, and cost effectiveness. However, generic sorbents (e.g., C18 or polymeric phases) provide limited selectivity, which may be inadequate for complex matrices under zero-tolerance requirements. In this context, MOF-based sorbents provide high surface area, tunable porosity, and versatile interactions, enabling faster mass transfer and enhanced adsorption compared with conventional SPE; nonetheless, their selectivity toward CAP relies mainly on physicochemical interactions rather than true molecular recognition, which can limit performance in highly complex matrices [30,103].

In this context, MIP SPE has emerged as a particularly promising selective clean-up strategy. Tailor-made recognition sites confer high selectivity for CAP, reduce co-extracted interferents, and effectively mitigate matrix effects. Coupled with LC–MS/MS, MIP SPE enables reliable quantification well below MRPL/RPA and has been successfully applied to milk, honey, meat, fish, and environmental waters [56].

Solid-phase microextraction (SPME) minimizes solvent use and integrates sampling, extraction, and preconcentration in a single step. However, its application to CAP is still limited by fiber selectivity and extraction efficiency in complex matrices. Early studies in biological matrices demonstrated feasibility, but with sensitivity and robustness inferior to SPE-based methods [104]. More recently, sophisticated SPME strategies coupled to LC–MS/MS and signal amplification schemes, such as enzyme-assisted SPME combined with a DNA nanowalker, have achieved very low detection limits in animal-derived foods [105].

Despite these advances, such methods rely on multi-step designs and advanced recognition elements, which currently limit their suitability for routine confirmatory analysis under zero-tolerance frameworks. Consequently, SPME is best regarded as a complementary or exploratory tool rather than a standalone solution for regulatory confirmation of CAP residues.

## 7. Conclusions and Future Perspectives

The determination of CAP residues in food and environmental matrices remains a major analytical challenge due to the compound’s strict regulatory prohibition, extremely low minimum required performance limits (MRPL), and the complexity of biological and environmental matrices. As highlighted throughout this review, substantial progress has been achieved over the past decade in both screening and confirmatory analytical methodologies, driven by the need for highly sensitive, selective, and reliable detection at trace and ultra-trace levels.

Screening methods, including immunoassays (ELISA and LFIA), biosensors, aptamer-based assays, and MIP-based sensors, have shown remarkable improvements in sensitivity, speed, and portability. These techniques are particularly valuable for high-throughput screening and on-site applications, allowing rapid identification of potentially contaminated samples. Nevertheless, their inherent limitations in specificity, susceptibility to matrix interferences, and potential cross-reactivity necessitate confirmatory analysis using chromatographic–mass spectrometric methods. Emerging biosensing technologies, particularly those integrating nanomaterials, surface-enhanced Raman spectroscopy (SERS), and electrochemical transduction, demonstrate promising sensitivity and portability. However, issues related to reproducibility, standardization, robustness, and large-scale validation remain significant barriers to widespread routine implementation.

Among confirmatory techniques, LC–MS/MS remains the undisputed gold standard for CAP determination. Its superior selectivity, sensitivity, and regulatory compliance allow reliable quantification at sub-ng/kg levels across a wide range of matrices, including milk, honey, seafood, meat, and environmental samples. Triple quadrupole instruments operating in multiple reaction monitoring (MRM) mode provide excellent quantitative performance, robustness, and reproducibility, making them the preferred choice in routine regulatory laboratories. High-resolution mass spectrometry, such as Orbitrap and QTOF systems, offers additional advantages such as retrospective data analysis, improved confidence in analyte identification, and the ability to perform multi-residue and non-target screening. However, despite these advantages, HRMS still faces limitations in routine regulatory application due to higher costs, complex data processing, and, in some cases, lower quantitative robustness compared to triple quadrupole systems.

Sample preparation continues to play a critical role in achieving reliable trace-level detection. Techniques such as QuEChERS and solid-phase extraction (SPE) remain widely used due to their balance between efficiency, reproducibility, and compatibility with chromatographic systems. Recent advances in microextraction techniques, including dispersive liquid–liquid microextraction (DLLME), fabric phase sorptive extraction (FPSE), and the use of advanced sorbents such as molecularly imprinted polymers (MIPs), metal–organic frameworks (MOFs), and nanostructured materials, have demonstrated improved selectivity, reduced solvent consumption, and enhanced enrichment factors. Despite these advances, challenges related to matrix effects, sorbent stability, method reproducibility, and scalability for routine laboratory use persist.

From a critical perspective, several important gaps remain in current analytical approaches. First, there is a need for greater harmonization and standardization of emerging screening technologies, particularly biosensors and nanomaterial-based platforms, to ensure reproducibility and regulatory acceptance. Second, while HRMS offers powerful capabilities for non-target analysis, further work is required to improve quantitative performance, simplify data analysis workflows, and establish standardized validation protocols. Third, matrix effects remain a persistent issue, particularly in complex food and environmental samples, highlighting the need for improved sample preparation strategies and the development of more robust internal standardization approaches. Furthermore, most current methods focus on targeted CAP detection, whereas the simultaneous monitoring of CAP metabolites, transformation products, and co-occurring contaminants represents an important but underexplored area.

Future research directions should focus on the development of integrated analytical workflows that combine rapid screening with highly reliable confirmatory techniques. The integration of miniaturized, portable, and automated systems with advanced detection platforms offers significant potential for on-site monitoring and real-time analysis. The application of novel functional materials, including advanced nanomaterials, hybrid sorbents, and selective recognition elements, may further improve extraction efficiency and analytical performance. Additionally, advances in data processing, including machine learning and chemometric approaches, may enhance signal interpretation, improve detection reliability, and facilitate large-scale monitoring programs.

In parallel, increasing emphasis should be placed on the development of environmentally sustainable analytical methods in accordance with green analytical chemistry principles, including reduced solvent consumption, simplified workflows, and reusable materials. Finally, continued collaboration between analytical scientists, regulatory agencies, and instrument manufacturers will be essential to ensure that emerging analytical technologies meet regulatory requirements and can be successfully implemented in routine monitoring programs.

Overall, while LC–MS/MS remains the cornerstone of confirmatory CAP analysis, ongoing advances in high-resolution mass spectrometry, biosensing technologies, and innovative sample preparation approaches are expected to further enhance analytical performance, improve monitoring efficiency, and support global efforts to ensure food safety and environmental protection.

## Figures and Tables

**Table 1:** Overview and comparison of analytical methods for CAP determination in food and environmental matrices.

Analytical Method	Typical Matrices	LOD	Selectivity	Main Advantages	Main Limitations	Regulatory Suitability	Ref.
Immunoassays (ELISA, lateral flow tests)	Food (milk, honey, fish, shrimp, meat…), water	pg–ng/mL; ng/kg	Medium	Rapid, low cost, high throughput; suitable for screening	Cross-reactivity, false positives, limited quantitative accuracy	Screening only	[26,27,28,29,30,31,32,33,34,35,36,37,38,39]
Biosensors	Food, water	ng/kg	Medium, high	Fast response, portable, minimal sample preparation	Limited robustness, matrix effects, still under development	Screening/confirmatory	[40,41,42,43,44,45,46,47,48]
SERS-based methods	Honey, milk, meat, water	pg–ng/kg	High	Ultra-high sensitivity; label-free detection; rapid analysis	Signal variability; complex substrate fabrication; limited standardization	Advanced screening	[49,50,51]
HPLC–UV/DAD	Food, meat, milk	µg/kg; <ng/mL	Low, high	Simple instrumentation, low cost	Insufficient sensitivity for zero tolerance; poor selectivity: high sensitivity when assisted by emerging microextraction strategies (MIL-LLME; MNF-DLLME; FPSE; MOF-SPE)	Not suitable, limited	[52,53,54,55,56]
HPLC–FLD (after derivatization)	Pharmaceutical formulations	ng–µg/kg	Medium	Improved sensitivity compared to UV	Derivatization required, time-consuming	Limited	[57]
GC–MS	Meat, fish, honey	ng/kg	High	Good separation and sensitivity	Derivatization required; thermal instability issues	Confirmatory	[58,59,60]
LC–MS/MS	Food (meat, milk, honey), environmental (aquatic biota, soil), urine	ng/kg or lower	Very high	High sensitivity and specificity; reference confirmatory technique	High cost, skilled personnel required	Confirmatory (gold standard)	[61,62,63,64,65,66,67,68,69,70,71]
UHPLC– Orbitrap HRMS	Aquatic foods, meat	mg/kg	Medium	Good for fast routine analysis	Results require confirmation	Screening	[22,72,73,74]
UHPLC– Orbitrap HRMS	Fish, water	µg/kg	Low, high	Not very specific and does not yield satisfactory results. Sample clean-up improves performance	Fast, suitable for routine analysis, laborious	Confirmatory	[75]
UHPLC–QTOF HRMS	Chicken, honey, cosmetics, urine	ng/kg	Very high	High sensitivity and specificity	High cost, skilled personnel required	Confirmatory	[20,76,77,78,79]

**Table 2:** Matrix composition and recommended sample preparation strategies for CAP analysis.

Matrix Type	Dominant Components	Main Analytical Challenges	Recommended Sample Preparation Strategies	Suitability for Zero-Tolerance Control
Milk	Proteins, lipids	Protein precipitation, ion suppression	LLE; QuEChERS; salting-out; selective SPE	High
Meat	Lipids, proteins	Lipid co-extraction, matrix effects	QuEChERS; SPE (PSA, C18, HLB); selective SPE	High
Eggs	Proteins, lipids	Protein precipitation, lipid interference	LLE; ASE; QuEChERS; SPE	High
Honey	Sugars, viscosity	Sugar interference, matrix effects	Salting-out extraction; MIP-SPE; selective SPE	Very high
Fish/ Seafood	Lipids, pigments	Pigments, fat content	QuEChERS; hybrid clean-up; selective SPE	High
Water	Low organic load	Low analyte concentration	Direct injection; SPE preconcentration	High
Soil/ Sediment	Organic matter	Adsorption, heterogeneity	Modified QuEChERS; solvent extraction; SPE	Moderate–High

**Table 3:** Critical comparison of sample preparation techniques for CAP determination.

Technique	Selectivity	Matrix Effect Reduction	Advantages	Limitations	Suitability for Regulatory Confirmation
LLE	Low	Limited	Simple, low cost	Poor selectivity, high solvent use	Limited
QuEChERS	Medium	Moderate	Fast, inexpensive, multi-residue	Limited clean-up for ultra-trace analysis	Good screening
Conventional SPE	Medium	Good	Robust, widely available	Limited specificity	Suitable
MIP-SPE	High	Excellent	High selectivity, low interference	Higher cost, target-specific	Excellent
Magnetic sorbents	High	Excellent	Rapid separation, high efficiency	Limited availability	Excellent
SPME	Low–Medium	Limited	Solvent-free, simple	Limited robustness	Exploratory

## Data Availability

No new data were created or analyzed in this study. Data sharing is not applicable to this article.
